# Gossypiboma‐associated sarcomas in five cases

**DOI:** 10.1111/jsap.70083

**Published:** 2026-01-26

**Authors:** G. Thomas, L. Doeven, A. Guillén, D. Brockman, E. Herbert, S. Priestnall, J. Helm, K. Shimura, M. Simpson

**Affiliations:** ^1^ Department of Clinical Science and Services The Royal Veterinary College Hatfield UK; ^2^ Department of Pathobiology and Population Sciences The Royal Veterinary College Hatfield UK; ^3^ Vets Now Referrals Glasgow UK; ^4^ Veterinary Referral Hospital Dandenong Dandenong Victoria Australia

## Abstract

**Objectives:**

To report clinical presentation, treatment and outcome of dogs and cats diagnosed with gossypiboma‐associated sarcoma.

**Materials and Methods:**

Referral centres within the United Kingdom, Europe, North America and Australia were contacted to search medical records for dogs and cats with documented gossypiboma‐associated sarcoma and included if comprehensive clinical records, histopathology and follow‐up to death were available.

**Results:**

Four dogs and one cat were included. The surgical procedures where the surgical gauze was accidentally retained were ovariohysterectomy (*n* = 4) and caesarean section (1). Time between this surgery and gossypiboma‐associated sarcoma diagnosis ranged from 1054 to 2201 days. Presenting clinical signs included anorexia (*n* = 5), lethargy (3), vomiting (2) and abdominal pain (2). Preoperative imaging documented an abdominal mass with no evidence of metastases in all cases. Surgical procedures to facilitate mass excision included duodenectomy (*n* = 1), partial pancreatectomy (1), partial gastrectomy (1), omentectomy (1) and ovariohysterectomy (1). Histopathology results were consistent with gossypiboma‐associated fibrosarcoma (*n* = 2), extraskeletal osteosarcoma (1), hemangiosarcoma (1) and poorly differentiated sarcoma (1), with all demonstrating chronic granulomatous inflammation with intralesional refractile fibre foreign material. One received adjuvant chemotherapy postoperatively. Lesions consistent with metastasis were identified on imaging in three dogs and one cat and suspected in one dog, between 11 and 114 days postoperatively. Survival time from surgery for cases 1 to 5 was 181, 56, 66, 13 and 196 days, respectively.

**Clinical Significance:**

Gossypiboma‐associated sarcomas are rare and exhibit aggressive clinical behaviour with a generally poor prognosis. Use of radiopaque surgical gauzes, surgical checklists and surgical gauze counts are imperative to prevent such neoplasms.

## INTRODUCTION

Major intraoperative complications are relatively infrequent, yet they can result in significant morbidity. While many complications are preventable, they may still occur despite adherence to standard surgical protocols, often due to human error. One such complication is a retained surgical gauze or gossypiboma (Kiernan et al., 2008). Gossypiboma is a term derived from the Latin word gossipium, meaning cotton and describes a granulomatous inflammatory lesion caused by a surgical gauze that has been accidentally left in a body cavity following surgery (Reed & Gosling, 2021). Synonyms for gossypiboma include textiloma, gauzoma or muslinoma and may be used instead to reflect the synthetic materials used in the current day (Deschamps & Roux, 2009). Gossypiboma‐related clinical signs may be absent for years, making diagnosis challenging (Auger et al., 2019), but fatal consequences can occur in human and veterinary patients alike (Dinler et al., 2017; Gawande et al., 2003). The incidence of gossypibomas in both human and veterinary medicine is unknown, but is thought to be low, with fewer than 60 cases reported in veterinary literature (Reed & Gosling, 2021). However, this complication is likely underreported due to potential unwanted criticism attached to accepting the human error involved (Kiernan et al., 2008). Despite recommended routine safety precautions such as surgical checklists, surgical gauze counts and use of surgical gauzes with radiopaque markers, this avoidable error has not been completely eradicated.

Gossypibomas have been documented in various locations in veterinary patients, including intrathoracic (Reed & Gosling, 2021), intra‐abdominal (Brun et al., 2021; Forster et al., 2013; Louvet & Duconseille, 2017; Rodriguez et al., 2018) and caudal to the proximal tibia (Corbin et al., 2013). The interval between the primary surgery where the surgical gauze was accidentally retained and discovery of the gossypiboma can vary from days to years and is often an incidental finding (Forster et al., 2013). Diagnosis of a gossypiboma can be made with radiography, ultrasonography, computed tomography (CT) and magnetic resonance imaging (MRI) (Mai et al., 2001; Merlo & Lamb, 2000). However, they are often only definitively diagnosed through histopathological examination following surgical excision.

Gossypibomas can mimic the appearance of neoplasia on imaging. This is well reported in human medicine (Haidari et al., 2023; Puvanesarajah et al., 2019) and has been reported twice in veterinary medicine. Corbin et al. (2013) documented a suspected implant‐associated sarcoma in a dog after a tibial plateau levelling osteotomy (TPLO). On histopathology, this was a gossypiboma alone. Deschamps and Roux (2009) documented a gossypiboma mimicking the appearance of a bladder neoplasm in a female neutered dog presenting for severe haematuria.

Rarely, gossypibomas can be associated with malignant neoplastic transformation. The pathogenesis of these tumours is complex and poorly understood in humans but is hypothesised to be associated with chronic inflammation and tissue injury induced by retained foreign materials. This may promote genetic mutations within adjacent soft tissues and impair local immune surveillance (Samargandi, 2024). This has been reported infrequently in human medicine and in only seven single case reports in veterinary literature (Goto et al., 2022; Haddad et al., 2010; Miller et al., 2006; Pardo et al., 1990; Peace & Riggs, 2014; Rayner et al., 2010; Slovak et al., 2015). The first report by Pardo et al. (1990) documented a primary jejunal osteosarcoma in association with a surgical gauze. The first report in a cat by Haddad et al. (2010) documented an abdominal gossypiboma‐associated fibrosarcoma. Other case reports include an extraskeletal osteosarcoma following TPLO (Miller et al., 2006), abdominal fibrosarcoma in two animals (Peace & Riggs, 2014; Rayner et al., 2010) and extraskeletal osteosarcoma in two animals (Goto et al., 2022; Slovak et al., 2015).

The aim of this study was to report the first case series of gossypiboma‐associated sarcomas in dogs and cats, including their presentation, treatment and clinical outcome.

## MATERIALS AND METHODS

This multi‐institutional retrospective observational study used anonymised clinical data. Forty‐one referral centres were contacted worldwide to include the United Kingdom, France, Italy, the Netherlands, Australia and North America. Each centre was asked to search the hospital medical records to identify dogs and cats diagnosed with a gossypiboma‐associated neoplasm prior to December 2024. To be included, it was required that each case record contained a comprehensive clinical history, histopathology confirming gossypiboma‐associated malignancy and follow‐up to death. For cases that met the inclusion criteria, databases searched included VetCompass, Helix, Rx Works Veterinary Software and AT Veterinary Systems. Search terms included gossypiboma, textiloma, gauzoma, muslinoma, gossypiboma‐associated, gossypiboma‐associated sarcoma/ neoplasm, surgical gauze associated sarcoma/ neoplasm, swab associated sarcoma/ neoplasm, retained swab, retained surgical gauze and gossypiboma transformation. Information retrieved from the medical records included signalment, date and type of initial surgery where a surgical gauze was accidentally retained, onset and type of clinical signs at diagnosis, haematology and biochemistry results, imaging findings, presence of metastasis pre‐ or postoperatively, surgery performed, histopathology, complications, adjunctive therapies, time to metastasis and survival time. All cases were deceased at the time of data collection; therefore, no owners were contacted for follow‐up.

## RESULTS

### Presentation

Four dogs and one cat met the inclusion criteria. All were female and four were neutered. The breeds were domestic short hair (DSH) (*n* = 1), Maltese terrier (1), Jack Russell terrier (1), Cocker spaniel poodle crossbreed (1) and Labrador (1). The previous abdominal surgical procedures performed included: ovariohysterectomy (OVH) in three dogs and one cat and caesarean section in one dog. All these surgeries took place at the referring veterinary practice and were the only previously documented abdominal surgeries performed. A complication was recorded in the medical records in case 3 during OVH, where significant haemorrhage was noted, and in case 5 during OVH where an incorrect postoperative surgical gauze count was recorded. For case 2, OVH was recorded as routine. For cases 1 and 4, the clinical notes for OVH and caesarean section, respectively, could not be obtained. The time between OVH or caesarean section and onset of first clinical signs related to the gossypiboma‐associated neoplasm was known in four cases and ranged from 1030 to 2194 days. The clinical signs included anorexia (*n* = 5), lethargy (3), vomiting (2) and abdominal pain (2). Other clinical signs included diarrhoea (*n* = 2), pyrexia (2), coughing (1) and weight loss (1). Age of presentation varied from 3 years and 9 months to 10 years and 3 months. Haematological abnormalities on presentation included neutrophilia (*n* = 3), leucocytosis (2), non‐regenerative anaemia (1), regenerative anaemia (1), eosinophilia (1) and monocytosis (1). Biochemical abnormalities on presentation included elevated creatine kinase activity (*n* = 2), elevated amylase activity (2), elevated alkaline phosphatase activity (2), elevated aspartate transaminase activity (1), hypoalbuminemia (1) and increased C‐reactive protein (1). The time between OVH or caesarean section and diagnosis of gossypiboma‐associated neoplasm ranged from 1054 to 2201 days.

### Imaging

**FIG. 1:**
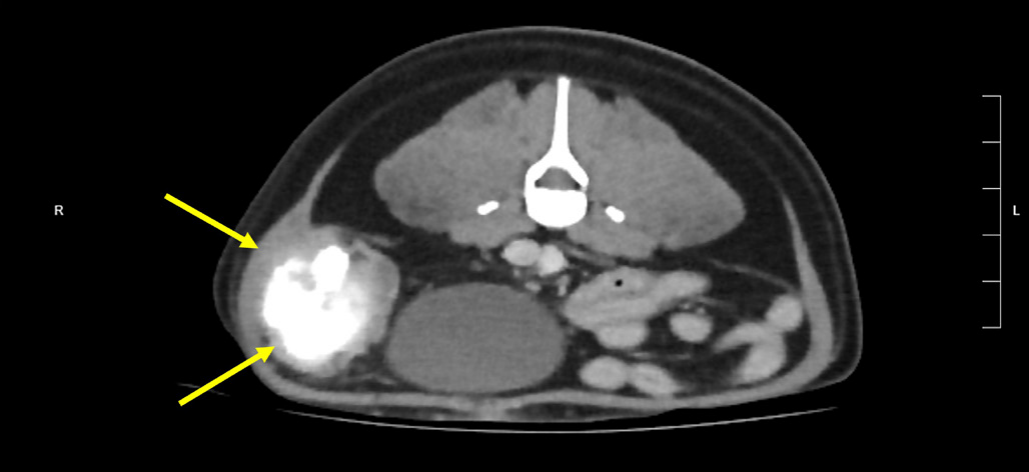
Demonstrating a large 3.6 cm by 3.1 cm (height by width) markedly hyperattenuating mass in the right mid to caudal abdomen (yellow arrows). Histopathology confirmed a gossypiboma‐associated extraskeletal osteosarcoma.

Preoperative imaging modalities used to identify the gossypiboma‐associated neoplasm were radiography (*n* = 3), ultrasonography (2) and CT (2). The imaging findings for case 1, utilising abdominal ultrasound and thoracic radiographs, revealed loss of abdominal serosal detail and a right‐sided abdominal mass. Imaging of the thorax was unremarkable. For case 2, utilising bicavitary CT, a 3.6 cm markedly hyperattenuating mass in the right mid to caudal abdomen was evident (Fig 1). Imaging of the thorax was unremarkable.

**FIG. 2:**
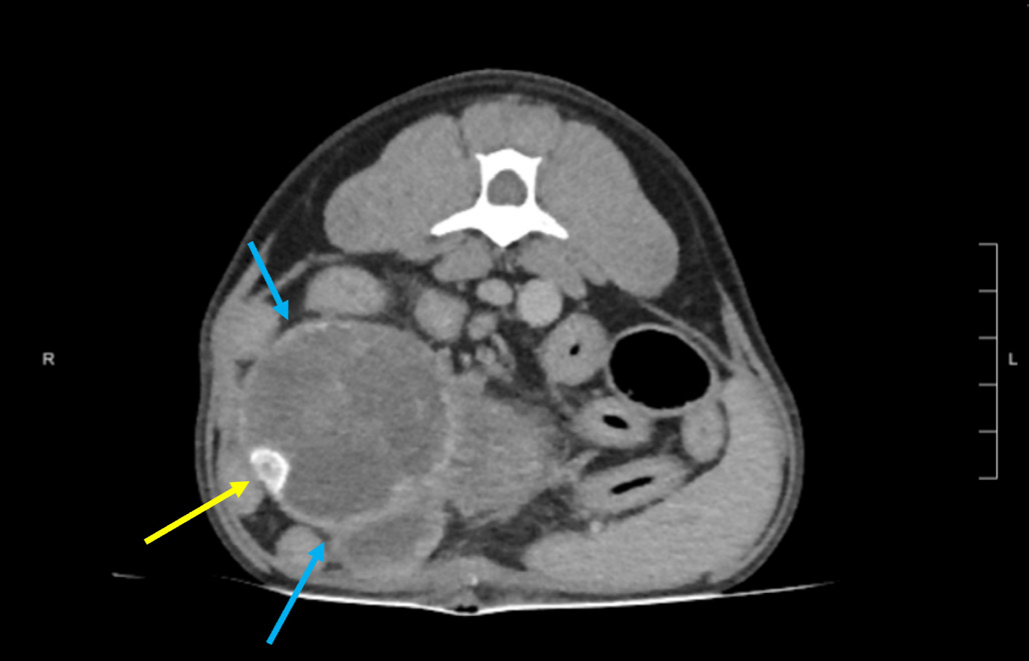
Demonstrating a large bilobed soft tissue attenuating mass in the mid to cranial abdomen (blue arrows) with foci of mineralisation towards the periphery of the mass (yellow arrow). Histopathology confirmed a gossypiboma‐associated fibrosarcoma.

For case 3, utilising bicavitary CT, a large bilobed soft tissue attenuating mass in the mid to cranial abdomen extending to the right of midline was identified, blending with the greater curvature of the stomach in the distal gastric body (Fig 2). Foci of mineralisation were noted towards the periphery of the mass. Imaging of the thorax was unremarkable.

Case 4 was diagnosed at the referring practice using abdominal radiographs. This documented an abdominal mass near the spleen with peritoneal effusion. No imaging of the thorax was performed. Case 5 was also diagnosed at the referring practice with abdominal ultrasound, documenting a right‐sided ventral heterogeneous abdominal mass with a small volume of surrounding abdominal free fluid. Radiographs of the thorax were unremarkable.

### Surgery

Abdominal laparotomy was performed in all cases. Intraoperative tumour locations were right mid‐caudal abdomen (*n* = 4) and left mid‐abdomen (1). Three out of five tumour excisions were performed in a referral hospital. No dog or cat had documented metastasis at the time of surgery. Concurrent surgical procedures to facilitate excision of the mass included duodenectomy (*n* = 1), partial pancreatectomy (1), partial gastrectomy (1), OVH (1) and partial omentectomy (1). No intraoperative complications were reported. The length of postoperative hospitalisation ranged from 1 to 5 days.

### Histopathology

**FIG. 3:**
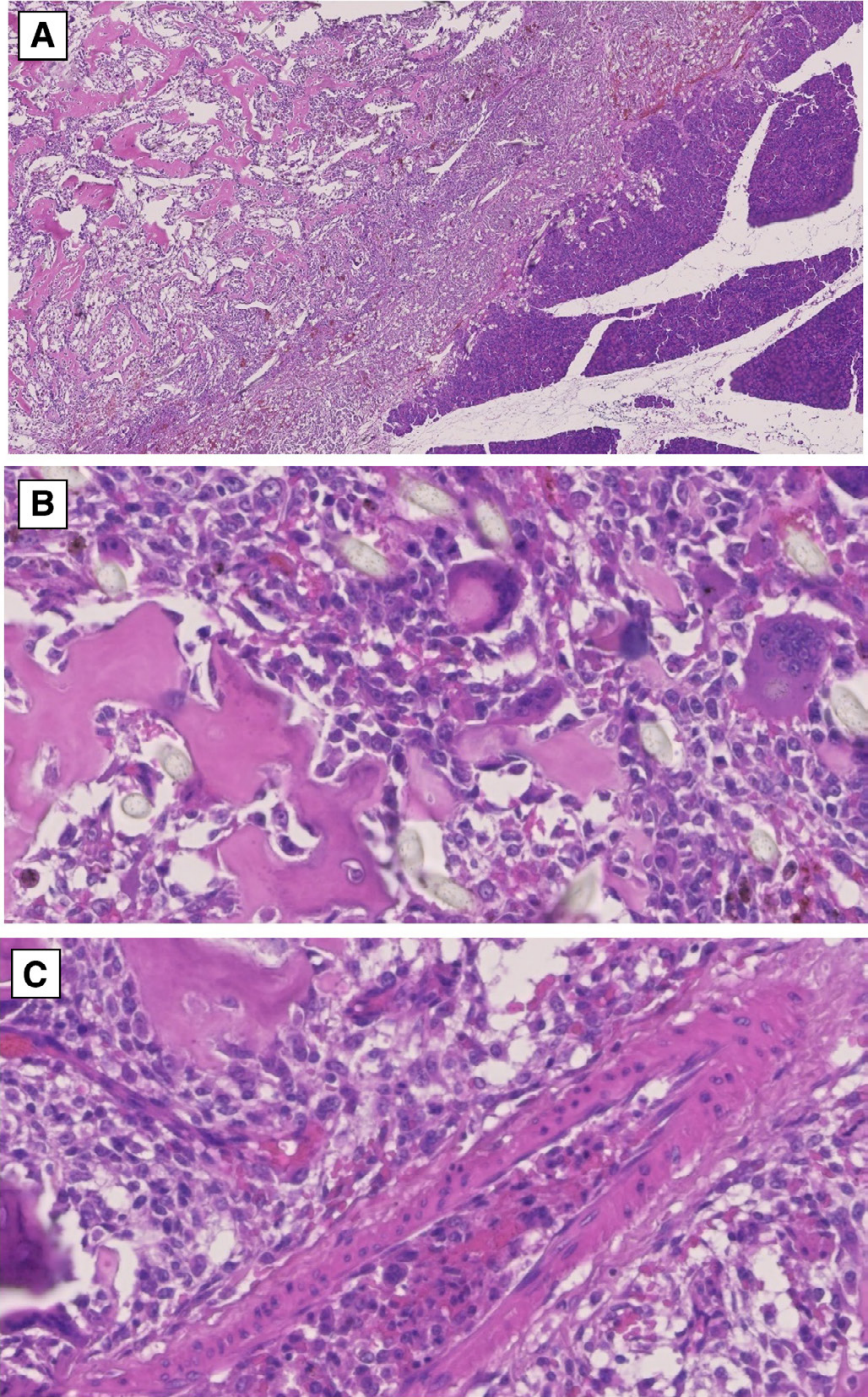
(A) Osteosarcoma adjacent to pancreas ×100. Mesenchymal neoplasm producing islands and trabeculae of eosinophilic matrix (osteoid) infiltrating pancreas. Bar = 100 μm. HE. (B) Fibres within multinucleated giant cells ×400. Neoplastic spindle cells surround foci of eosinophilic homogeneous to fibrillar matrix (osteoid) and multinucleated giant cells engulf refractile material. Bar = 25 μm. HE. (C) Intravascular tumour cells within the blood vessel lumen ×400. Bar = 25 μm. HE.

**FIG. 4:**
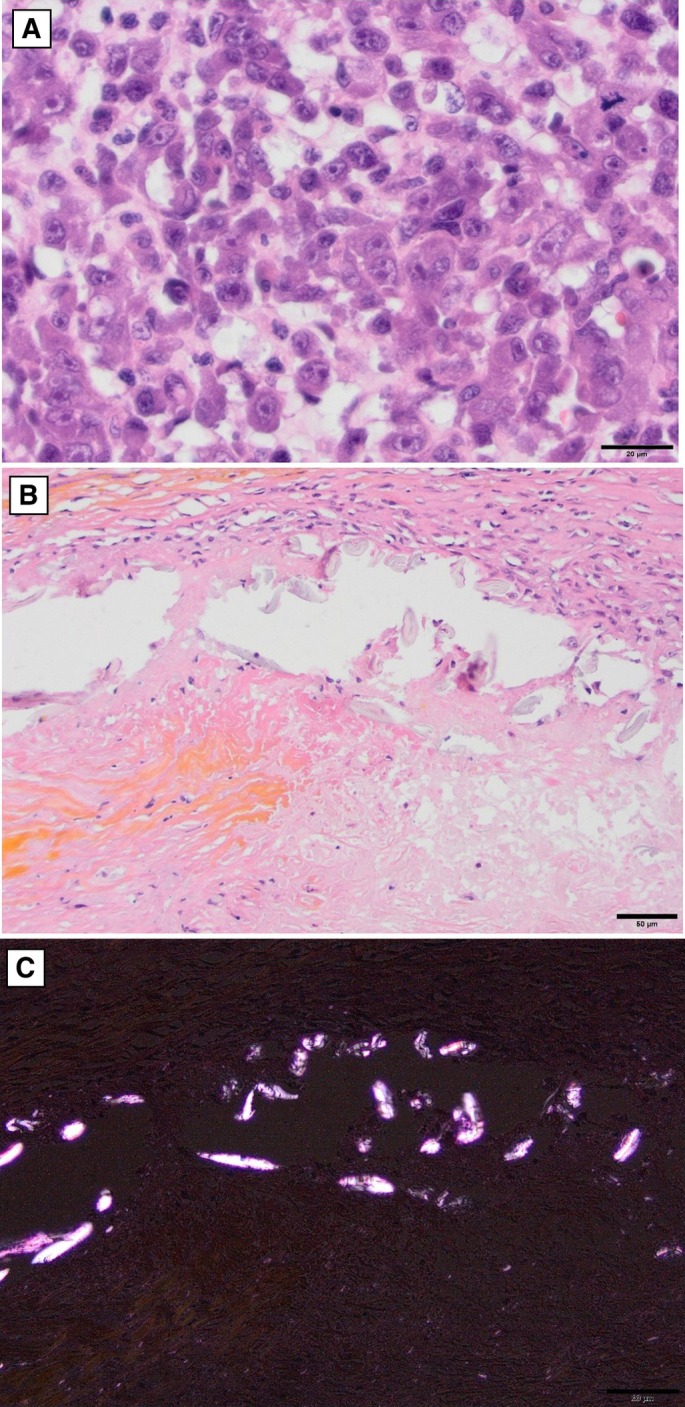
(A) Fibrosarcoma ×600. Loosely arranged streams of plump and highly pleomorphic neoplastic fibroblasts with marked anisocytosis and anisokaryosis. Bar = 20 μm. HE. (B) Foreign material ×200. Fragments of refractile material embedded within organising haemorrhage, collagen and chronic inflammation. Bar = 50 μm. HE. (C) Foreign material (gauze fibres) under polarised light ×200. The same field (as above) viewed under polarised light demonstrating the refractile nature of the material.

Histopathological diagnoses included gossypiboma‐associated osteosarcoma (*n* = 1) (Fig 3), fibrosarcoma (2) (Fig 4), hemangiosarcoma (1) and poorly differentiated sarcoma (1). In all cases, histopathology identified foreign material consistent with cotton from a surgical gauze with chronic granulomatous inflammation and occasional phagocytosis adjacent to the sarcoma. Evidence of occasional phagocytosis indicated true presence of the foreign material as opposed to postoperative contamination of the samples. In case 4, due to the poorly differentiated cell morphology, an immunohistochemistry panel including antibodies to vimentin, cytokeratin, Wilms Tumour‐1, thrombomodulin, calretinin, Factor 8, and Iba‐1 was performed. All markers showed negative immunolabelling, and the sarcoma could not be further characterised.

### Outcome and follow‐up

All cases survived to discharge. No postoperative complications were reported. Adjunctive chemotherapy with doxorubicin was performed in one dog (case 3). After two doses, the dog was presented for the third planned dose but had deteriorated rapidly, with dyspnoea, abdominal distention, marked weight loss and hyporexia. Thoracic and abdominal ultrasound were performed and revealed bicavitary effusion, a large heterogeneous mass within the mid‐abdomen, and marked irregularities within the spleen, liver and kidneys consistent with diffuse metastasis or possible sarcomatosis. The dog was euthanised. Case 1 and 5 were treated palliatively with tapering prednisolone (1 mg/kg per os once daily to 0.5 mg/kg per os every other day over 2 weeks) and meloxicam (0.1 mg/kg per os once daily), respectively. In cases 2 and 4, adjunctive therapy was offered but declined by the owner.

Lesions consistent with metastasis were identified postoperatively with imaging in three dogs and one cat, utilising bicavitary ultrasound (*n* = 2) and bicavitary CT (2). Disease‐free interval was 114 days in case 1 (cat) and 66, 11 and 28 days in cases 3 to 5 (dogs), respectively. Disease‐free interval was unknown for case 2 as the dog was euthanised prior to imaging. Average time to metastasis was 55 days. Location of metastasis was liver (*n* = 2), pulmonary (2), cranial mesenteric lymph node (2), spleen (1), kidneys (1), sternal lymph node (1), diaphragm (1), omentum (1), body wall (1) and retroperitoneal with invasion of the caudal vena cava (1).

Survival from surgery was 181 days in case 1 (cat), 56, 66, 13 and 196 days in cases 2 to 5 (dogs), respectively. All deaths were due to, or suspected to be due to, gossypiboma‐associated sarcoma metastasis. In cases 1, 3, 4 and 5, euthanasia was performed due to severe deteriorating quality of life or collapse secondary to documented metastasis. In case 2, the dog was presented to the referring veterinarian for deteriorating quality of life. The dog was euthanised at the owner’s request. No imaging was performed to confirm metastasis, although highly suspected as no other disease processes had been documented and histopathology of the gossypiboma‐associated sarcoma had found intravascular invasion with neoplastic cells within a blood vessel. One dog underwent postmortem (case 4), which revealed gossypiboma‐associated disseminated metastatic neoplasia throughout the liver, lung, lymph nodes, diaphragm, omentum and body wall. Pertinent case details are included in Table 1 to include signalment, initial surgery type, clinical signs at presentation, time between initial surgery and diagnosis, primary care or referral surgery, neoplasm location, concurrent surgical procedure to facilitate excision of gossypiboma‐associated sarcoma, histopathology, adjunctive therapy, disease‐free interval and survival from surgery.

**Table 1:** Signalment, initial surgery type, clinical signs at presentation, time between initial surgery and diagnosis, primary care or referral surgery, neoplasm location, concurrent surgical procedure to facilitate excision of gossypiboma‐associated sarcoma, histopathology, adjunctive therapy, disease‐free interval, survival from surgery in four dogs and one cat diagnosed with gossypiboma‐associated sarcoma

Case	Signalment at presentation	Initial surgery	Presenting clinical signs	Time between initial surgery and diagnosis of gossypiboma‐associated neoplasm (days)	Primary care or referral surgery	Location of neoplasm	Concurrent surgical procedure to facilitate excision	Histopathology	Treatment	Disease‐free interval (days)	Survival from surgery (days)
1	9 years and 2 months FN, DSH	OVH	Vomiting, anorexia	Unknown	Referral	Right mid‐caudal abdomen	Duodenotomy	Gossypiboma‐associated fibrosarcoma	Prednisolone	114	181
2	5 years and 9 months FN Maltese terrier	OVH	Vomiting, anorexia	1901	Referral	Right mid‐caudal abdomen	Partial pancreatectomy	Gossypiboma‐associated extraskeletal osteosarcoma	None	Not confirmed	56
3	10 years and 3 months FN Jack Russell terrier	OVH	Anorexia, lethargy	2201	Referral	Right mid‐caudal abdomen	Partial gastrectomy	Gossypiboma‐associated fibrosarcoma	Doxorubicin every 3 weeks	66	66
4	7 years FE Labrador	Caesarean section	Anorexia, lethargy	1822	Primary care	Left mid‐abdomen	OVH	Gossypiboma‐associated poorly differentiated sarcoma	None	11	13
5	3 years and 9 months FN Cocker spaniel poodle X	OVH	Anorexia, lethargy	1054	Primary care	Right mid‐caudal abdomen	Partial omentectomy	Gossypiboma‐associated hemangiosarcoma	Meloxicam	28	196

FE Female entire, FN Female neutered, OVH Ovariohysterectomy

## DISCUSSION

In this case series, we have documented five cases in which surgical gauzes were accidentally retained following routine abdominal surgery and malignant tumours arose in association with the retained gauze many years later. In these dogs and cats, the evidence that retained surgical gauzes were instrumental in tumorigenesis is compelling. To obtain these cases, the authors contacted forty‐one referral centres worldwide, who each searched their medical database for dogs and cats diagnosed with gossypiboma‐associated sarcomas. Despite this extensive search, only four dogs and one cat were identified, demonstrating the apparent rarity of these events. An OVH was the only previous surgery in three dogs and one cat, and a caesarean section in one dog. Therefore, we could be certain of when the surgical gauze was accidentally retained in the abdomen. The prognosis for these cases was poor, with survival following surgical removal of the gossypiboma‐associated malignancy ranging from 13 to 196 days.

Four of the initial surgeries where the surgical gauze was accidentally retained were routine OVHs. This is consistent with previous studies where OVH was the most common procedure associated with an accidentally retained surgical gauze (Forster et al., 2013; Rodriguez et al., 2018). Previously identified risk factors for accidentally retained surgical gauzes in human medicine include emergency operations, unplanned changes in the surgical procedure and high body mass index (Gawande et al., 2003; Lata et al., 2011). Additional risk factors include poor communication between the surgical team, length of surgery, patient instability, intraoperative blood loss, number of surgeries performed by the same team, inadequate staffing and inexperienced staff (Lata et al., 2011; Moffatt‐Bruce et al., 2014). In veterinary patients, previously identified risk factors for retained surgical gauzes include the absence of a defined and scheduled time for surgery, low number of staff involved in the surgical procedure and inadequate methods of surveillance. Methods of surveillance are imperative and currently include the use of radiopaque marker surgical gauzes, surgical gauze counting and surgical checklists (Rodriguez et al., 2018). Given the routine nature of OVH surgeries and the high frequency with which they are performed, there may be reduced willingness or perceived need to complete a surgical gauze count due to the seemingly low risk of surgical gauze retention. Thus, the type of surgical procedure may influence the likelihood of a retained surgical gauze.

In three dogs and one cat, the surgical gauze was retained during OVH and this led to a right‐sided gossypiboma‐associated sarcoma. Exteriorisation and ligation of the right ovarian pedicle are known to be more challenging (Bowlt et al., 2011), as the right ovary is situated further cranial compared to the left. During OVH, haemorrhage from the right ovarian artery has been shown to be the most common complication (Bowlt et al., 2011; Burrow et al., 2005). We would anticipate that most veterinary surgeons stand on the right side of the dog or cat during abdominal surgery, given that the majority of the population are right‐handed. As such, visualising an intra‐abdominal blood‐soaked surgical gauze packed onto the right ovarian pedicle may be more challenging. Therefore, the position of the surgeon and the location of the intra‐abdominal surgical gauze may increase the likelihood of retention. To prevent this, the side of the dog or cat closest to the surgeon should be thoroughly examined prior to closure and a surgical gauze count performed.

A notable complication was documented in the surgical notes of two OVH procedures. In case 3, the ligature slipped on the right ovarian pedicle and significant haemorrhage was noted. The site was packed with surgical gauzes, the pedicle was located and re‐ligated, with successful haemostasis achieved. In case 5, there was an incorrect postoperative surgical gauze count with only four surgical gauzes counted out, where five surgical gauzes were counted in. Despite extensive exploration, the final surgical gauze was not located. As the surgical gauzes utilised did not contain a radiopaque marker, the abdomen was closed and the owner was notified. It is plausible that significant intraoperative complications, such as haemorrhage or a rushed surgical closure due to patient instability, may increase the risk of accidentally leaving a surgical gauze in situ. This case also highlights the absolute requirement for use of radio opaque surgical gauzes during surgery. For the remaining three cases, one OVH was recorded as routine, and the clinical notes of the remaining causative surgeries could not be obtained.

Metastasis was documented in cases 1, 3, 4 and 5 at 114, 66, 11 and 28 days, respectively, after excision of the gossypiboma‐associated sarcoma. Average time to metastasis was 55 days. Case 2 was euthanised 56 days postoperatively due to poor quality of life and suspected metastasis. This compares to previous literature by Pardo et al. (1990) and Haddad et al. (2010), documenting time to metastasis of 60 days and Miller et al. (2006) documenting time to metastasis of 90 days. This demonstrates that gossypiboma‐associated sarcomas are consistently aggressive and carry a poor prognosis. Case 4 was diagnosed with metastasis only 11 days following excision of the gossypiboma‐associated sarcoma and was euthanised 13 days postoperatively. This dog underwent abdominal radiography prior to surgery at the referring veterinarian’s practice. The dog recovered poorly from surgery and was referred for further investigations five days postoperatively. Bicavitary CT demonstrated both pulmonary and abdominal metastasis. It is therefore considered extremely likely that metastases were present at the time of surgery, but not documented due to absence of thoracic imaging, poorer sensitivity of radiography to detect small metastatic lesions compared to CT and possible human error. This dog presented multiple times to the referring veterinarian prior to surgical exploration and so the dog may have undergone investigations late in the disease process with metastasis already present. This is considered the reason for the markedly shorter survival time compared to the other four cases.

Investigation into the process of gossypiboma‐associated neoplastic transformation was not performed during this study, nor researched in other veterinary literature. However, other neoplasms share a common feature of an initiating event. Examples include vaccine administration in the case of an injection site sarcoma, trauma in the case of fracture‐associated sarcoma (Atherton & Arthurs, 2012; Boudrieau et al., 2005) or a foreign body, such as glass fragments or microchips, leading to the development of a sarcoma (Carminato et al., 2011; McCarthy et al., 1996; Vascellari et al., 2006). Another well‐described phenomenon, with pathogenetic similarities to foreign body‐induced neoplasia in dogs and cats, is oesophageal fibrosarcoma or osteosarcoma. This may be induced in dogs infected with *Spirocerca lupi*, by parasite‐specific antigenic stimulation and intense granulomatous inflammation associated with the parasite. Although the exact pathogenesis of this has not been determined (Ranen et al., 2004). In gossypiboma‐associated neoplasms specifically, the cotton fibres of the surgical gauze and the microenvironment created by the fibrous capsule have been proposed as factors in sarcomatous transformation of gossypibomas (Miller et al., 2006). In humans, numerous cases of malignant transformation, particularly angiosarcomas, have been reported at the site of a retained surgical gauze or other foreign material such as an internal cardiac defibrillator implant (Smith et al., 2013). Future research into the pathogenesis of gossypiboma‐associated sarcomas is warranted.

Prevention of these avoidable neoplasms is vital. A surgical checklist and surgical gauze count should be undertaken for every surgical procedure. The World Health Organization has shown that implementing a standardised checklist can reduce inpatient complications from 11% to 7% (Haynes et al., 2009). A surgical gauze count was recorded in the clinical notes of only one of the reported cases; the remainder made no comment of a surgical gauze count being performed. Little evidence is available on the effectiveness of current counting processes; however, it remains the only inexpensive method available and must be undertaken with care and diligence. One human study showed the accuracy of counting surgical gauzes had a sensitivity of 77% and a specificity of 99%, with a retained surgical gauze occurring in 1 of 7000 procedures (Egorova et al., 2008). Long surgical procedures, late procedures and changes in nursing personnel throughout the procedure were risk factors. Incorrect manual counts were noted in 63% to 88% of cases of retained foreign objects in human medicine (Weprin et al., 2021). In one veterinary study, 26.6% of respondents did not perform a surgical gauze count (Rodriguez et al., 2018). Surgical gauzes should be separated, counted audibly and concurrently viewed during the count procedure by two individuals. Rodriguez et al. (2018) showed that 56.3% of veterinarians used non‐radiopaque surgical gauzes. It was not possible to identify the type of surgical gauze used in this small cohort, except in case 5 where non‐radiopaque surgical gauzes were utilised. Use of radiopaque surgical gauzes is imperative as well as performing postoperative radiographs if an incorrect surgical gauze count is encountered. A potential consideration is performing routine postoperative radiographs for high‐risk surgeries. In human medicine, it has been suggested that all high‐risk cases, such as people undergoing bariatric and emergency surgical procedures, should have routine postoperative radiography to detect any retained items (Mahran et al., 2013). However, this was not proven to be a cost‐effective method (Egorova et al., 2008). Additionally, it would increase the number of radiographs and therefore radiation exposure by 14.6 times compared with a policy recommending radiographs only for count discrepancies (Egorova et al., 2008). Advanced approaches include the use of bar‐coded surgical gauzes which have been shown to improve discrepancies in counting and misplaced surgical gauzes (Greenberg et al., 2008). Additionally, surgical gauzes with radiofrequency identifying tags have been experimentally investigated and showed an improved efficiency in the surgical gauze counting process (Lazzaro et al., 2017). However, at present, these methods are unlikely to be available or economically viable for the veterinary profession.

The small sample size of this case series leads to inherent limitations. With only four dogs and one cat, this was descriptive only and no statistical comparisons were possible. We are unable to state that this is truly representative of the population. However, as the databases of forty‐one referral centres were searched, it is evident that these are extremely rare cases. Our study was retrospective; as such, there was no standardisation in preoperative diagnostic work up, surgery, postoperative care, staging or adjunctive therapy. We were reliant on medical records alone for reporting these cases. Although extremely rare, our study documents only dogs and cats referred to referral hospitals for further treatment. Therefore, cases could have been missed if not referred and managed by the referring veterinarian. Additionally, these four dogs and one cat presented between 1030 and 2194 after the surgical gauze was accidentally retained. Due to the long interval between retention and associated clinical signs, such cases could have been lost due to death from other causes or have unknowingly died as a result of gossypiboma‐associated neoplasm. In addition, given the difficult nature of dealing with these cases, it is possible that they are underreported to avoid potential criticism. Further research into the occurrence of such cases in general veterinary practice is warranted.

In conclusion, this case series documents the rare occurrence of gossypiboma‐associated sarcomas in dogs and cats. These tumours consistently exhibited aggressive clinical behaviour leading to a poor prognosis and short survival times. Use of radiopaque surgical gauzes, surgical checklists and surgical gauze counts are imperative to prevent such neoplasms.

### Author contributions

**G. Thomas:** Conceptualization; data curation; writing – original draft; writing – review and editing; methodology; investigation; visualization. **L. Doeven:** Data curation; writing – review and editing; investigation. **A. Guillén:** Conceptualization; writing – review and editing; supervision. **D. Brockman:** Conceptualization; writing – review and editing; supervision. **E. Herbert:** Data curation; writing – review and editing. **S. Priestnall:** Data curation; writing – review and editing. **J. Helm:** Data curation; investigation; writing – review and editing. **K. Shimura:** Data curation; writing – review and editing. **M. Simpson:** Conceptualization; writing – original draft; writing – review and editing; methodology; supervision; investigation.

### Conflict of interest

The authors declare no conflict of interest.

## Data Availability

The data that support the findings of this study are available from the corresponding author upon reasonable request.
